# The relationship of gender with attitudes toward physical activity, level of physical activity, and aerobic capacity among medical students

**DOI:** 10.3389/fpsyg.2025.1645209

**Published:** 2026-01-12

**Authors:** Hajar Al-Rajaibi, Khoula Al-Toubi, Said Aljaadi, Sanjay Jaju, Deepali Jaju

**Affiliations:** 1 Department of Physiology, Sultan Qaboos University, Muscat, Oman; 2 Department of Clinical Physiology, Sultan Qaboos University Hospital, Muscat, Oman; 3 Oman Medical Speciality Board, Al Athaiba, Oman; 4 Department of Family Medicine and Epidemiology, Sultan Qaboos University College of Medicine and Health Sciences, Muscat, Oman

**Keywords:** IPAQ-SF, Chester Step Test, Kenyon scale, medical students, physical activity, aerobic capacity

## Abstract

**Introduction/Objective:**

Counseling about physical activity (PA) by doctors is influenced by their own attitudes towards physical activities and personal aerobic capacity levels. The study aims to explore ‘gender-specific relationship’ in Attitude Towards PA (ATPA), self-reported PA levels and Aerobic Capacity levels among senior medical students.

**Methods:**

In this cross-sectional study (*n*=110; males 55; age 21.39 SD 0.939 years), ATPA was measured using a fifty-four-items Kenyon questionnaire; and self-reported PA level using International Physical Activity Questionnaire-Short Form (IPAQ-SF). The estimated aerobic capacity level was measured using Chester step test (CST).

**Results:**

Both genders showed a positive overall Kenyon score for ATPA (Females: Males 3.31:3.33). Females scored higher in the domains of health and fitness (4.15 *vs* 3.75, *p*<0.001); and reduction of tension (3.59 *vs* 3.32, *p*<0.012) whereas males scored higher in the domains of pursuit of vertigo (2.71 *vs* 3.06, *p*=0.001) and in tension and risk (2.7 *vs* 2.94, *p*=0.011). Estimated aerobic capacity using CST showed that males had (48.37 SD 10.11) higher score than females (40.35 SD 5.77; *p*<0.001; mean difference 8.02). IPAQ-SF analysis categorized 64% students having moderate level of PA but it did not correlate with estimated aerobic capacity measurement using CTS.

**Conclusion:**

Despite both genders having an equally positive overall ATPA, females prioritized health and stress reduction, while males value thrill and risk. The gap between attitude and PA behavior needs tailored, gender-specific intervention strategies.

## Introduction

Regular physical activity reduces the risk of non-communicable diseases, poor physical and cognitive function, and mental ill-health.(Bull et al., 2020). Physical inactivity (PIA) is considered as one of the major modifiable risk factors for chronic diseases. Katzmarzyk estimated prevalence-based population attributable risks for 168 countries and documented that globally, PIA is a cause of 7.2% of all-cause deaths and 7.6% of deaths due to cardiovascular disease (Katzmarzyk et al., 2022). A pooled analysis of 507 population-based surveys with 5·7 million participants published in the Lancet reported that the global age-standardized prevalence of insufficient physical activity (PA) was 31·3% in 2022 which 23.4% higher than in the year 2000 and 26·4% higher than in 2010 (Strain et al., 2024). The physical inactivity-related healthcare costs associated with non-communicable diseases were estimated to be $53.8 billion worldwide in 2013, of which $31 billion was paid by the public sector (Ding et al., 2016).

Promoting PA through physical activity counseling is an effective intervention to address this high global burden of physical inactivity. The policy of the World Health Organization (WHO) is to incorporate PA into health services and social services to reduce global physical inactivity by 15% by the year 2030 (Bull et al., 2020). Inclusion of PA counseling in chronic diseases management can have a significant impact in reducing morbidity and mortality of these chronic diseases (Blair et al., 1995). The importance of PA counseling has been addressed over past 3 odd decades. Different studies have shown that the PA counseling is low (Alghafri et al., 2017; Glasgow et al., 2001; van der Ploeg et al., 2007) and it could be attributed to different barriers like limited availability of time with patients, lack of knowledge about guidelines/standards, lack of training in prevention of diseases, lack of facilities and inadequate skills for effective counseling (Alghafri et al., 2017; Lawlor et al., 1999).

Physicians have an ethical obligation to provide PA advice to their patients (Snyder and for the American College of Physicians Ethics, Professionalism, and Human Rights Committee*, 2012). Physicians are considered as the most credible and respected source information related to health, which position them the best to provide PA counseling, feedback and follow-up (Lobelo et al., 2009). However, the barriers to PA counselling exist which includes insufficient training and motivation of doctors and improvable personal PA habits.

Medical students, even though they are aware of the benefits of physical activity, may not meet the recommended level of physical activity (Dąbrowska-Galas et al., 2013). Gender differences in physical activity and attitude toward PA have been documented. Male students tend to exercise for relaxation, quality of life, and female students generally for weight loss and toning (Seo and Ha, 2019; Craft et al., 2014).

To date, no study in Oman has explored the attitudes of medical students toward physical activity, assessed their physical activity, or measured their aerobic capacity levels. Hence, there is a need to target future health professionals in Oman to generate evidence to strengthen current health promotion strategies in combating the menace of chronic diseases in Oman. In addition, since all Omani citizens receive free curative healthcare services from the government, the findings of this study could be meaningfully utilized to devise methods to save the financial losses for the country due to the large amounts incurred on the treatment of illnesses. This study is conducted with two objectives: (1) to explore “gender-differences” (i) in attitude toward PA; (ii) in self-reported PA levels; and (iii) in aerobic capacity levels among senior medical students by using standardized instruments; and (2) to understand the “gender-specific” relationships between (i) attitude toward PA and self-reported PA; (ii) attitude toward PA and aerobic capacity; and (iii) self-reported PA and aerobic capacity.

## Methods

This is a cross-sectional study conducted at the Department of Clinical Physiology of Sultan Qaboos University Hospital (a tertiary care Hospital). The research was approved by the Research Ethics Committee of the Medical College (MREC #1814), and informed consent was obtained from every participant.

The study sample consisted of phase II medical students at College of Medicine and Health Sciences, Sultan Qaboos University (COM&HS, SQU), as they have exposure to medical subjects and their knowledge about health is expected to be relatively better compared to students of phase one. The MD program at this medical college comprises three phases, which lead, when completed successfully, to the award of an MD degree in a minimum of 6 years. Phases II and III represent the medical component of the medical program. The phase II is of 4 semesters and focuses on advanced study of normal human structure and function; pre-clinical subjects; and sociological, epidemiological, ethical, and scientific aspects of medical practice through early medical contact.

The sample size was calculated for a desired confidence level of 95% and an absolute precision of 10% with the assumption that at least 50% of the medical students would have the desired level of both physical activity and a positive attitude toward physical activity. This required studying 96 students to study. For the assumption that only 20% would be tested, fit on objective evaluation, the required number for the study is 61. The higher estimate of 96 students plus an additional 10% was considered appropriate for this study (CMC Biostatistics, n.d.) A total of 293 students were registered in the phase II in years 2019-20 out of which 110 students (males 55) were randomly selected for the study from among those who volunteered to participate and were found eligible.

The purpose of this research was explained via an announcement in each class by the researchers. From a pool of 293 students that are registered in phase II in the year 2019–20, out of which 110 students (55 men) were randomly selected for the study from among those who volunteered to participate and were found eligible. Students having a cold, illness, or taking beta-blocker medicines were excluded, as these factors will affect the test performance of the Chester Step Test (CST). All the subjects signed a written informed consent, which included the study details. The schedule of participants for investigations in this study was prepared based on their availability from the academic program in COM&HS. Each subject required approximately 2 h to complete the questionnaires and the fitness test; hence, only 4 students were studied each day.

## Data collection

### Attitude toward physical activity measurement

A validated Arabic version of the Kenyon ATPA questionnaire was used to measure the attitude toward physical activity (Al-Rawahi and Al-Yarribi, 2013). A total of 54 items under the following six domains, health and fitness, reduction in tension, esthetic, social experience, pursuit of vertigo, and tension and risk, were assessed. Health and fitness mean “interest in health and fitness.” Reduction in the tension means that “participants believe that physical activity releases life’s tension and stress and makes them relax.” Esthetic experience “is the beauty and creative qualities that certain physical activities portray, for example, gymnastics.” In Social experience, the students believe “that physical activity is beneficial to them as it enhances their social lives; this means that they manage to socialize with people in their surroundings, and this makes their lives better.” Pursuit of vertigo refers to “the thrill that results from the body being disoriented when people get involved in activities that involve quick direction change, speed, acceleration, or exposure to situations that are dangerous.” Tension and risk mean that students believe “that physical activities ensure that they do not encounter tension and risks.”

Five-point Likert scale (strongly agree, agree, no idea, disagree, and strongly disagree) was used. All positive items were scored one for strongly disagree and five for strongly agree, while all negative items were scored five for strongly disagree and one for strongly agree (Al-Rawahi and Al-Yarribi, 2013).

### Physical activity level measurement

The self-reported IPAQ-SF was used, which provided subjective physical activity data for the last 7 days. Physical activity information was collected on three domains, which are activity at work, travel from place to place, and recreational activity. The activity is documented at four intensity levels: vigorous-intensity activity, moderate-intensity activity, walking, and sitting. Physical activity was then categorized based on total MET-min/week as low (<600 MET-min/week), moderate (at least 600 MET-min/week), or high (at least 3,000 MET-min/week) (Craig et al., 2003).

### Aerobic capacity using the Chester Step Test (CST)

Chester Step Tests. The CST is an objective test that provides a prediction or estimation of maximum oxygen consumption (VO2max) through extrapolation, and not a direct measurement (which would require gas exchange analysis). To highlight this subtle but important distinction for methodological precision, we have used the term ‘aerobic capacity’ throughout the manuscript for estimated VO_2max_ / predicted VO_2max_ tests require minimal equipment, are easy to administer in limited indoor spaces, and can be administered by personnel with little or no formal training in exercise physiology. The CST is a valid test for the estimation of aerobic capacity (Sykes and Roberts, 2004). This submaximal, multistage test consists of five stages, each lasting for 2 min.

Students were asked to abstain from eating and drinking tea, coffee, alcohol, and smoking for at least 2 h before the test. They were asked not to exercise for 1 day prior to testing to ensure a constant baseline activity level. Anthropometric measurements, height, weight, and waist circumference, were measured before conducting the test. Resting heart rate was recorded. The 80% of the maximum heart rate (220-age) was calculated.

During CST, the subject is asked to step on a 24-cm height step to a metronome rhythm given using a mobile metronome app. Level 1 of the CST starts with 15 steps/min for 2 min. The frequency of stepping is increased by 5 steps per min for each stage. At the end of each stage, heart rate was recorded using a heart rate monitor (Myzone MZ-3, China) and level of exertion recorded using the Borg scale (0–20; 0 no exertion at all; 20 maximum exertion). The test continued until the participant reached 80% of the predicted maximum heart rate or ceased upon the participant’s request. The maximum test duration is 10 min (stage 5).

Aerobic capacity of each participant is predicted by plotting the exercise heart rates against their corresponding stage. A best-fit line was drawn between the plotted points, and the line was extended up to the maximum HR. The meeting point between the best-fit line of the HR readings and the maximum HR was used to determine the aerobic capacity (mlO_2_/kg/min) from the x-axis.

### Statistical analysis

The continuous variables were presented as mean (SD), while the categorical variables were presented as frequencies and percentages. The normality of the continuous variables was determined by the Kolmogorov–Smirnov test, and based on this, an independent samples t-test or Mann–Whitney test was used to analyze the quantitative data, as appropriate, to test the significance of the difference between male and female students. Chi-squared test was used to assess the association of gender with level of physical activity and with aerobic capacity, and also to determine the gender-specific association between IPAQ-SF categories and CST categories.

The “gender specific” aspect has been studied separately for male students and female students by (a) domain-wise correlation of attitude toward physical activity as measured by scores on the Kenyon ATPA questionnaire with (i) PA as measured in METs per min per week, and (ii) aerobic capacity mlO2/kg/min. (b) Association between PA and aerobic capacity. A *p*-value of less than 0.05 was considered significant.

## Results

The mean age of the medical students was 21.39 (SD 0.939). Male medical students had significantly higher age, waist circumference, and BMI compared to female students (Table 1).

**Table 1:** Characteristics of students (*n* = 110).

Demographic characteristics		Men (*n* = 55)	Women (*n* = 55)	*P*
		Mean (SD)	Mean (SD)	*t*-test
Waist Circumference (cm)		82.80(10.39)	72.89 (9.35)	*p* < 0.001
BMI (Kg/m^2^)		24.74 (3.74)	22.78 (4.11)	*p* = 0.010
		n (%)	n (%)	Chi-squared test
BMI categories	Underweight	3 (5.5)	8 (14.5)	*p* = 0.021
	Normal	22 (40.0)	31 (56.4)	
	Overweight	27 (49.1)	12 (29.8)	
	Obese	3 (5.5)	4 (7.3)	

BMI, body mass index.

In the gender difference in attitude toward physical activity (ATPA) measurement (Table 2), the mean attitude of female students is significantly higher in the domain of health and fitness (*p* < 0.001) and reduction in tension (*p* < 0.012). However, in the domain of pursuit and vertigo (*p* = 0.001) and in tension and risk (*p* = 0.011), the male students had a significantly higher mean score (Table 2). The ranking in the different domains was nearly similar among both male and female students, except in the domains of reduction in the tension and esthetic experience. There was no significant gender difference in overall Kenyon scores.

**Table 2:** Gender differences in attitudes toward physical activity as measured by subjective responses using the Kenyon ATPA questionnaire.

Domains	Men		Women		*t*-test *p*-value
	Mean (SD)	Order	Mean (SD)	Order	
Health and fitness	**3.74 (0.62)**	1	**4.15 (0.44)**	1	**<0.001**
Reduction of the tension	**3.32 (0.63)**	3	**3.59 (0.48)**	2	**0.012**
Esthetic experience	3.41 (0.70)	2	3.20 (0.66)	3	0.097
Social experience	3.28 (0.68)	4	3.14 (1.03)	4	0.405
Pursuit of vertigo (Thrill)	**3.06 (0.63)**	5	**2.71 (0.45)**	5	**0.001**
Tension and risk	**2.94 (0.47)**	6	**2.70 (0.50)**	6	**0.011**
Overall Kenyon score	3.33 (0.34)		3.31 (0.25)		0.810

Bold number indicates *p*<0.05.

Table 3 highlights that the majority of the students (36 men and 34 women) fall into a moderate level of physical activity. Male-to-female ratio in the low PA was 7:15, and proportion of male students was two times higher in the high level PA (12:6). There was no significant gender difference in physical activity levels as measured by subjective responses using IPAQ-sf.

**Table 3:** Gender differences in physical activity levels as measured by subjective responses using IPAQ-sf.

Level of physical activity*	Men (n)	Women (n)	Chi-squared test *p*-value
Low (<600 MET-min/week)	7	15	0.830
Moderate (at least 600 MET-min/week)	36	34	
High (at least 3,000 MET-min/week)	12	6	

*The physical activity levels are obtained by converting the IPAQ scores into METs/min/week and then categorized into three categories (low, moderate, and high) (21).

The gender difference in aerobic capacity level as measured by CST showed a higher proportion of female students fall within the average to below average categories, while a lower proportion demonstrates excellent levels as compared to male students (*p* = 0.02). The mean (SD) aerobic capacity in males is 48.37 (10.11) is significantly higher than that in females 40.35 (5.77) (*p*<0.001, 95% CI 4.914, 11.133) (Table 4).

**Table 4:** Gender differences in aerobic capacity levels objectively measured using the Chester Step Test.

Level of aerobic capacity	Frequency (percent %)		Chi-squared test *p*-value
	Men	Women	
Below average	1 (1.8)	6 (10.9)	**0.020**
Average	16 (29.0)	21 (38.2)	
Good	28 (51.0)	26 (47.3)	
Excellent	10 (18.2)	2 (3.6)	
Total	55 (100)	55 (100)	

Bold number indicates *p*<0.05.

The gender specific correlation (Table 5) between domain-wise Attitude towards physical activity (subjective) and Physical Activity as measured by IPAQ-SF (subjective) showed weak but significant correlations as follows: In males in reduction of tension (*r*=0.39, *p*=0.003) and in pursuit of vertigo (*r*=0.34, *p*=0.11) while in females in social experience (*r*=0.27, *p*=0.042). In overall Kenyon scores, we noted moderate level of correlation in males (*r*=0.50, *p*=0.006) and females (*r*=0.45, *p*=0.010). The same table also demonstrates the gender specific correlation between domain-wise Attitude towards physical activity (subjective) and aerobic capacity as measured by CST (objective) in which weak but significant correlation was noted in males in health and fitness (*r*=0.35, *p*=0.009). Power analysis based on mean difference in aerobic capacity between males and females (0.999); and of ‘reduction of the tension’ in males (0.847) showed that the study was adequately powered, however, the power analysis based on correlation coefficient of ‘reduction of the tension’ in females (0.396); and of ‘social experience’ in males (<0.001) showed that the study was underpowered.

**Table 5:** Gender specific correlation between domain-wise attitude toward physical activity (subjective) and (A) physical activity as measured by IPAQ-SF (subjective); and (B) aerobic capacity as measured by Chester Step Test (objective).

Attitude toward physical activity domains (Kenyon ATPA questionnaire)	Physical activity METs per minute per week (IPAQ-SF)				Aerobic capacity mlsO_2_/kgm/min (Chester Step Test)			
	Males		Females		Males		Females	
	r_s_	Sig.	r_s_	Sig.	r_s_	Sig.	r_s_	Sig.
Health and fitness	0.04	0.765	−0.05	0.695	**0.35**	**0.009**	−0.18	0.187
Reduction of the tension	**0.39**	**0.003**	0.23	0.091	0.19	0.165	−0.13	0.347
Esthetic experience	0.25	0.065	0.23	0.091	−0.07	0.609	0.06	0.663
Social experience	−0.04	0.795	**0.27**	**0.042**	−0.01	0.938	−0.04	0.782
Pursuit of vertigo (Thrill)	**0.34**	**0.011**	0.26	0.050	0.07	0.631	0 08	0.569
Tension and risk	0.15	0.272	0.06	0.686	0.09	0.531	0.08	0.569
Overall Kenyon score	**0.50**	**0.006**	**0.448**	**0.010**	0.13	0.508	<0.00	0.983

Bold number indicates *p*<0.05.

Both gender-specific association and correlation between self-reported physical activity level (IPAQ) and level of aerobic capacity as measured by CST yielded non-significant results (Table 6).

**Table 6:** Gender specific association between self-reported physical activity level (IPAQ) and level of aerobic capacity as measured by CST

Aerobic capacity level (CST)	Self-reported physical activity categories (IPAQ)							
	Males			*P*-value*	Females			*P*-value*
	Low	Moderate	High		Low	Moderate	High	
Below average	1	0	0	*p*=0.086	1	1	0	*p*=0.604
Average	1	13	2		8	16	2	
Good	5	17	7		6	16	3	
Excellent	0	6	3		0	1	1	

*Chi square test.

## Discussion

This study finding of high overall mean scores on Kenyon ATPA questionnaire demonstrates that the attitude of our medical students toward participation in PA is definitely positive with nearly similar outcomes as compared to an earlier study in the same university conducted on physical education students (Al-Rawahi and Al-Yarribi, 2013). Positive attitudes towards PA have been documented among medical students in different settings using different attitudes scales (Frank et al., 2008; Holtz et al., 2013; Sahlqvist et al., 2022). This reflects the awareness and knowledge of our medical students about health, social and psychological outcomes of the physical activity and could be attributed to the growing public campaigns in the country to raise the awareness about importance of physical activity (Mabry et al., 2014).

However, we found clear gender differences in our study in the context of attitudes across the different domains studied. The positive attitude of females towards PA as compared to males is noted in their interest in health and fitness and their belief that PA releases their tension and stress and makes them relax. For males, the resulting ‘thrill’ and tension and risk during PA carries more value. Previous research found similar results where female students rated health, strength, endurance, appearance, revitalization, and stress management higher than male students and male students were more motivated by enjoyment, challenge, competition, and social factors (Ednie and Stibor, 2017). Therefore, addressing these motivational differences through gender-specific strategies is crucial for maximizing engagement and effectively utilizing the students’ initial positive attitudes. The interventions for female students should prioritize activities framed around well-being, mindfulness, and community support to mitigate academic stress and strategies targeting male students should target intrinsic motivators such as organized competitive sports leagues or achievement-based activities.

The majority of medical students in this study were found to have a moderate level of physical activity as measured with the self-reported IPAQ-sf instrument. In neighboring Gulf countries, more than 50% of Saudi medical students reported having a low level of physical activity (Abdel-Salam and Abdel-Khalek, 2016). Almost 50% of Kuwaiti college students reported themselves as physically inactive (Al-Isa et al., 2011). Our study indicated that Omani medical students reported themselves as physically more active compared to students in the other Gulf countries who have a similar sociodemographic profile. In fact, Omani medical students’ physical activity is closer to what has been reported by American and Canadian medical students, where more than half (61%) of U. S. medical students and 64% of the Canadian medical students met the physical activity international recommendations (Frank et al., 2008; Holtz et al., 2013).

Overall, a higher proportion of female students had lower aerobic capacity compared with male students. Studies on Brazilian college students (Mesquita et al., 2018) and Turkish college students (Taskin, 2016) showed similar results with a similar trend of lower aerobic capacity in female students compared to male students. Male students have more advantageous muscle mass, muscle fibers, type, dimension, and electromechanical aspects than female students, and they have a higher capacity for using glycogen than female students. This could explain the comparatively higher aerobic capacity in male students (24).

The gender-specific results showed a weak correlation between attitude towards physical activity for health and both self-reported PA level and the aerobic capacity. This gap is largely attributed to practical barriers that prevent students from translating intention into action. Lack of time to exercise was commonly reported as a major barrier to exercise due to heavy curriculum and lack of exercise facilities around their medical schools (Lobelo et al., 2009). Addressing these barriers through targeted interventions is essential to bridge the gap between attitude and behavior. Medical college policy makers can close the gap by recognizing gender-specific motivations shown in this study. For female students (motivated by health and relaxation), the college should integrate easy access to de-stressing PA. For male students (driven by thrill and competition), the focus should be on maximizing high-intensity engagement through quick, competitive intramural sports leagues and strength challenges. By tailoring the type of activity and the time available to these distinct needs, college can turn positive attitude into sustained action.

We did not find studies correlating IPAQ and the CST in the literature, however in our analysis, we found that the IPAQ-SF and CST correlations were not-significant and hence we cannot comment on the correlation coefficients, which, incidentally are very weak. The IPAQ has demonstrated weak to moderate correlations (Spearman’s ρ≈0.09−0.53) when validated against objective standards such as accelerometry and pedometers (Farshbaf-Khalili et al., 2021; Prince et al., 2008). It is important to note that these tools assess distinct constructs: the CST measures cardiorespiratory fitness, a physiological capacity, while the IPAQ-SF measures physical activity recall, which means they capture different aspects of physical activity. Furthermore, the IPAQ is known to frequently overestimate activity levels (Ekelund et al., 2006; Steene-Johannessen et al., 2016). Although the study population—young medical students—might be expected to have a clear recall of their physical activity over the past seven days, the risk of overestimation of results cannot be excluded. Consequently, combining objective and subjective PA assessment tools is recommended to enhance the validity of PA research outcomes.

## Limitations

This study sample consists of a convenience sample of volunteers from one medical college in Oman. Hence, the results and conclusions of the study should be viewed with caution due to the potential for selection bias (e.g., volunteers may be more interested in fitness than non-volunteers) in our study. This significantly limits the external validity and generalizability of the findings to all Omani medical students.

Another limitation of this study is difficulty in calculating sample size. We did not find any study comparing Attitude towards physical activity (ATPA) measurement; Physical activity level measurement using International Physical Activity Questionnaire-Short Form (IPAQ-SF) and Aerobic capacity using Chester step test (CST). One earlier study documented a large group of medical students (26%) who, despite being aware of benefits of physical activity, did not meet the recommended level of physical activity (Dąbrowska-Galas et al., 2013). Based on this, and since we did not have prior data, and due to climatic conditions in Oman along with the cultural/traditional restrictions on females, we assumed that around 50% of our medical students would not have the desired level of both physical activity and positive attitude. Out of these 50% who confess to undertaking PA based on subjective responses, we assumed that only 20% would actually be classified as fit on objective evaluation. A total of 293 students were registered in the phase II in years 2019-20 out of which 110 students (males 55) were randomly selected for the study from among those who volunteered to participate and were found eligible. A recent study estimated that, globally, only 25–40% of the university student population is involved in regular Physical Activity (Grujičić et al., 2022).

Power analysis based on correlation coefficient of ‘reduction of the tension’ in females; and of ‘social experience’ in males show that our study is underpowered, thus affecting the robustness of our conclusion.

## Conclusion

Studying the gender-specific barriers and motivational predictors for PA is essential for informing public health policies, allowing institutions to design targeted interventions that effectively reduce the burden of physical inactivity. Medical students in our study showed a positive overall attitude toward PA, but significant gender-specific motivational strategies required. Female students are strongly driven by PA for Health and Fitness and Reduction of Tension, necessitating wellness programs focused on stress mitigation and community. Conversely, strategies targeting male students should leverage their documented preference for competition, challenge, and achievement, ultimately enabling future clinicians to translate these tailored PA habits into a healthy, active life that directly reflects in the effective implementation of PA counseling for their future patients.

## Data Availability

The original contributions presented in the study are included in the article/supplementary material, further inquiries can be directed to the corresponding author.
